# An interview with Alvaro Alfredo Figueroa

**DOI:** 10.1590/2176-9451.20.4.026-031.int

**Published:** 2015

**Figure:**
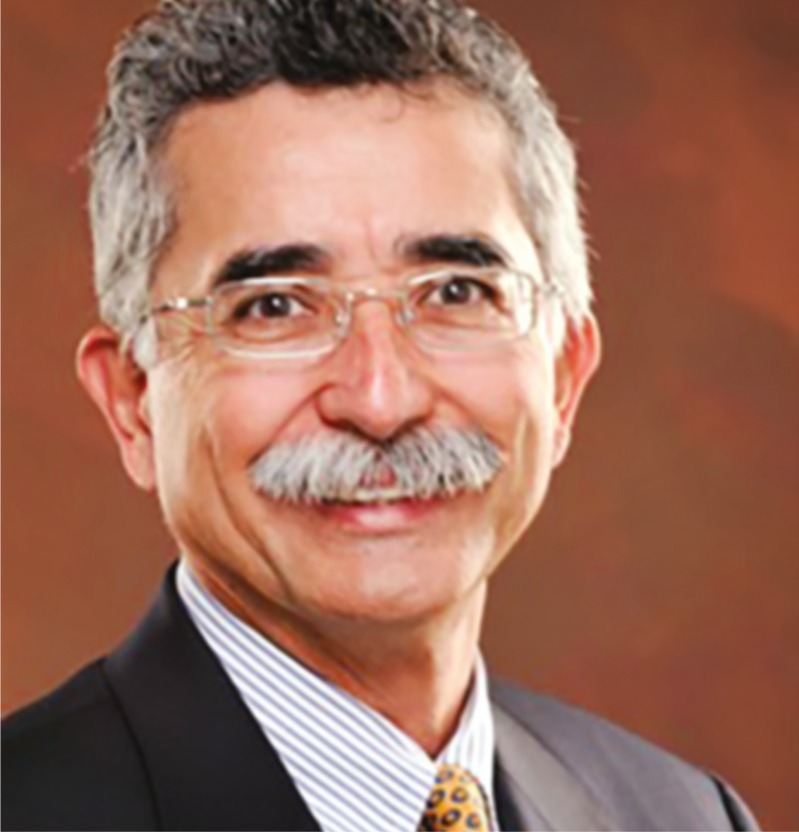

Alvaro Alfredo Figueroa grew up in Guatemala City, Guatemala, the son of a physician. He was always intrigued by the healthcare field and made it his life and livelihood when he attended dental school at the University of San Carlos in Guatemala. Shortly after graduation, he spread his wings and found a position researching at the National Institute of Health (NIH) in Bethesda, Maryland, USA. It is there that his passion for craniofacial anomalies and treatment developed. From NIH, he moved to Rochester, NY, where he had the good fortune of being an orthodontic resident at the Eastman Dental Center under the strict eye and tutelage of J. Daniel Subtelny. Dr. Subtelny's passion for orthodontic and surgical treatment of cleft lip and palate invigorated Alvaro who, after receiving his orthodontic specialty certificate in 1980, moved to Chicago, Illinois, to continue his studies. In Chicago, he attended the University of Illinois Chicago (UIC) and received his Pediatric Dentistry certificate and Master's degree. While working on his Master's research, he also took time to teach in the Department of Orthodontics and also began to play an integral role in the UIC Craniofacial Center. In 1999, he occupied to his current position at Rush University Medical Center as the Director of the Craniofacial Center. Over his more than thirty-year career, Alvaro has published a multitude of articles and textbook chapters. The Journal of Craniofacial Surgery awarded him the "Best Paper of the Year", in 1997, for his work on Distraction Osteogenesis, and in 1998, he received the highly coveted BF and Helen Dewel award from the American Journal of Orthodontics and Dentofacial Orthopedics for best clinical research paper. Dr. Alvaro is married to Michele and father of Alex, also an orthodontist, and Aaron, a maxillo-facial surgeon, a warm and united family passionate about Dentistry. It is with great pleasure and honor that I share with the readers my admiration for Dr. Alvaro Figueroa.

Dauro Douglas Oliveira

Alvaro Alfredo Figueroa é filho de médicos e cresceu na cidade da Guatemala, na Guatemala. Sempre interessado pela área de Saúde, ele entrou para a faculdade de Odontologia da University of San Carlos, na Guatemala. Recém-formado, assumiu o cargo de pesquisador no National Institutes of Health (NIH) na cidade de Bethesda, Maryland/EUA, onde sua paixão pelo tratamento das anomalias craniofaciais se desenvolveu. Do NIH, mudou-se para Rochester, NY, onde teve a honra de fazer residência em Ortodontia no Eastman Dental Center, sob a rigorosa orientação de J. Daniel Subtelny. A paixão do Dr. Subtelny pelo tratamento ortodôntico e cirúrgico de pacientes com fissura labiopalatina inspirou Álvaro, que, após especializar-se em Ortodontia, em 1980, mudou-se para Chicago, Illinois/EUA, para dar continuidade aos seus estudos. Em Chicago, frequentou a University of Illinois Chicago (UIC) onde se especializou em Odontopediatria e cursou o mestrado. Simultaneamente ao desenvolvimento de sua pesquisa de mestrado, ele também lecionou no Departamento de Ortodontia e dedicou-se ao UIC Craniofacial Center. Em 1999, assumiu seu atual cargo de diretor do Craniofacial Center do Rush University Medical Center. Ao longo de sua carreira de mais de 30 anos, publicou inúmeros artigos e capítulos de livros. O Journal of Craniofacial Surgery lhe concedeu o prêmio de "Melhor Artigo do Ano", em 1997, pelo seu trabalho sobre distração osteogênica; já, em 1998, ele recebeu o tão cobiçado B. F. and Helen Dewel Award, do American Journal of Orthodontics and Dentofacial Orthopedics, pelo melhor artigo de pesquisa clínica. Dr. Alvaro é casado com Michele e pai de Alex, ortodontista, e Aaron, cirurgião bucomaxilofacial: uma família amorosa e unida pela paixão à Odontologia. É com grande satisfação e honra que compartilho com os leitores a minha admiração pelo Dr. Alvaro Figueroa. 

Dauro Douglas Oliveira

## The first case of monobloc craniomaxillofacial distraction osteogenesis reported on the literature was performed by your team. How did you get involved with distraction? (Eduardo Franzotti)

In 1992, Dr. McCarthy and his group in New York introduced distraction to treat patients with hemifacial microsomia (HFM)^1^ and soon after that our group started performing distraction on HFM patients. In Mexico City, Drs. Molina and Ortiz Monasterio^2^^,^ 3 expanded the use of distraction to treat other deformities. They applied distraction to treat patients with cleft lip and palate, performing distraction for maxillary advancement using a face mask with elastic traction. We tried their approach in a couple of patients, but it did not work as well for us because, on occasion, patients did not wear the elastics, control of the elastic force was not easy, and there was skin irritation over the chin as a result of the pressure we had to apply to advance the maxilla. 

Soon after Dr. McCarthy published his paper, we invited him to visit our unit in Chicago and he presented his experience on distraction and on treatment of craniofacial anomalies. At that time, there was an infant in the pediatric intensive care unit with a severe form of Pfeiffer syndrome. Due to the severity of the case, the need for ocular protection and constant around-the-clock nursing care, urgent care was required. However, traditional craniofacial surgery was not feasible due to the limited amount of bone around the skull; thus, we started planning how to use distraction to treat the patient, since it had not been done before. My craniofacial surgery colleague, Dr. John Polley, and myself developed a special crib for the baby. The crib had out riggers that reminded "face bows", and distraction screws were incorporated to do the advancement. After performing monobloc osteotomy cuts, two horizontal metal fixation plates were placed above each orbit to prevent perforation of the thin bone by traction wires. In addition, trans zygomatic wires were used, a total of four: two supraorbital and two in the malar infraorbital regions. The patient was successfully distracted, his eyes were protected and the skull shape changed from extreme brachycephaly to scaphocephalic. This was the first reported monbloc advancement with external distraction.^4^

## The treatment of patients with hemifacial microsomia is centered on the mandibular deformity, but surgical timing is still controversial. How do you select the right time for intervention? (Monica Tirre)

There is much debate on the timing of surgery for HFM patients, either operating in growing or non-growing patient. The severity of HFM varies widely and it is functional impairment what should dictate the timing of surgical intervention. Patients with respiratory distress and feeding issues are candidates for early intervention.

Mandibular elongation by gradual distraction can be done at any age. In a study to be published this year in the AJO-DO, we observed that adolescent patients in full permanent dentition with orthodontic appliances placed to align and level the arches prior to bimaxillary distraction had better results than patients in primary and early mixed dentition stages.

One must consider that acute changes in mandibular shape during unilateral mandibular distraction result in postoperative alterations in dental occlusion, such as open bite on the affected side, crossbite on the contralateral side and, on occasion, anterior crossbite. These consequences of mandibular distraction require complex orthodontic treatment with fixed appliances over a long period of time. Additionally, postoperative orthodontic management can be challenging in the young patient due to limited cooperation levels. Whenever possible, we prefer to use bimaxillary distraction during adolescence as initially described by Ortiz-Monasterio and Molina,^5^ from Mexico. This approach requires application of intermaxillary fixation during the distraction process. When performed with orthodontic alignment and appliances, it allows for improvement of both maxillary and mandibular asymmetries and eliminates the need for extensive post-operative orthodontics.

## Why do you perform osteotomy instead of corticotomy? (Monica Tirre)

The group in Mexico initially recommended the use of corticotomy, but, on occasion, the bone did not fracture as intended and patients had to be operated again to have bones separated and allow for distraction. In some instances, the distractors bent without elongating the bone. In order to avoid the uncertainty of the procedure, we prefer complete osteotomy. This allows one to test the ability of the distractor to open and separate the bone and it renders the procedure predictable. 

## The rigid external device (RED) for midface distraction that you and Dr. John Polley developed is widely used around the world. How did you develop it and what are its main advantages? (Geórgia Lau)

There were other clinicians applying distraction to treat craniofacial deformities, not only hemifacial microsomia, but clefts and also craniosynostosis, such as Crouzon and Apert syndromes. At that point, we thought of using an internal distractor after performing a monobloc surgery, and we had an internal distractor that we had designed. Through the coronal incision, we performed a monobloc operation and attached the internal distractor behind the zygomatic arch and on the temporal bone. The distractor had a posterior arm, or activating arm, that went through the scalp. We had a patient that we distracted and she did well, but when we needed to remove the distractors, it became very difficult because bone had grown over the distractor. We had to perform another operation and it was very difficult to have access and take the internal distractors out. 

Thus, based on that experience, we decided that we needed a distractor that was not submerged underneath the skin for application.^6^Dr. John Polley had experience with neurosurgery, particularly with putting on neurosurgery halos and removing them, as he did during his general surgical rotations, and I was very familiar with the use of the protraction orthodontic face mask. Therefore, we thought about using an external halo as an anchorage point. For me, it was relatively easy to develop a splint attached to the teeth that would pull the bone forward. The first case we treated, which is published in the journal of Craniofacial Surgery, had a fantastic outcome.^7^ The patient was a boy with bilateral cleft lip and palate with severe maxillary hypoplasia, secondary to the cleft. His change was unbelievable and this encourage us to continue with our approach. The main advantage of RED is that it requires a single operation, and, therefore you do not need a second one to remove the distractor. This device has a vertical arm that allows adjustment of force vectors of distraction anytime during the process; thus, controlling maxillary rotation. It also allows the surgeon to perform osteotomy as high as possible, since there is no need to do any fixation above and below it. That is not possible using an internal device, as sufficient bone is required to anchor the device above and bellow osteotomy. Fixation of an internal device has the risk of damaging dental roots, especially those of teeth that are partially erupted or unerupted.

The disadvantage is that RED is external. Since only severe patients use this protocol, with proper education and family support, the patients can accept the use of the appliance.

## You have been working with RED for over 20 years. Along these years, which were the main improvements of the appliance? (Lúcio Maia)

The design of the initial external traction hooks attached to the intraoral splint was a bit of a process. At the beginning, I soldered them by means of orthodontic 0.040 wire or 0.060-in heavy laboratory wire, but that was not strong enough. I then decided to use an external headgear and an inner bow to prepare the splint. Moreover, we also used a customized palatal arch around the perimeter of the arch to support the splint and make it more rigid. In addition to that, we soldered cantilever wires in 45^o^ to make the external traction hooks very rigid. The main problem with the headgear system was that, at the time of surgery, the patient had all these wires in front of the face and it was difficult for the anesthesiologist to manage the patient. I decided to find a way of doing removable external traction hooks. Currently, we have a splint that uses rectangular tubes, similar to those used in the Mara appliance, which receives the external traction hooks made of heavy and rigid rectangular wire. The intraoral splint is manufactured in the USA by an orthodontic laboratory company. The halo is produced commercially and there are two companies that manufacture it at this time. The system that we originally designed for cleft patients^6^was also used for craniofacial patients. Based on the experience of the baby, we pulled from four points (two supra orbital and two at the dental level through the intraoral splint traction hooks). In this way, we could control the rotation of the large monobloc bone segment.

## The rigid external device has been used in different craniofacial patients, such as clefts, Crouzon and Apert syndromes patients. Do you have a specific protocol for each craniofacial condition? (Geórgia Lau)

Latency, activation and consolidation protocols do not change based on the deformity of the patient, but on the severity of the maxillary or midface deficiency. Another difference is the type of osteotomy required to correct the deformity. Patients with Crouzon and Apert syndromes require a monobloc osteotomy while cleft patients usually require a high Le Fort I osteotomy. 

The latency period relies on the age of the patient. Younger patients require a shorter latency period, three to five days, whereas adolescents and adults have to wait five to seven days before activation. For the craniosynostosis cases, because they are very severe patients and most of the time younger, the latency period is short too. The rate of distraction does not vary, it is 1 mm per day, one turn in the morning and one turn at night. The amount of distraction depends on the deformity and plan required for each patient.

## Distraction osteogenesis has shown to be safe and efficient to treat craniofacial deformities. Could non-syndromic patients benefit from this procedure? (Lúcio Maia)

We can use distraction in non-syndromic or cleft patients. Once we became familiar treating very difficult craniofacial patients, we began to apply the technique to patients that had dental facial deformities. However, the majority of the cases are severe cleft and syndromic patients. In orthognathic surgery, surgeons have limits in how much they can advance the maxilla, so patients that require more than 8-10 mm of maxillary advancement are the ones that benefit from distraction. 

## It is known that during maxillary advancement, controlling the center of rotation of the maxilla is a challenge. Is it possible to control the line of action of distraction force with the RED appliance? (Eduardo Franzotti)

One possible side effect from maxillary advancement with distraction is the development of open bite. To prevent it, we pull from the front of the maxilla and not from the molar region. We also use a traction hook above to the palatal plane, so it is possible to control the rotation of the maxilla. Nanda had estimated the center of rotation of the maxilla to be at the apex of the first molar. Dr. Ahn, from South Korea, while in Chicago with our group, developed a model to determine where the center of rotation of the osteotomized maxilla was, and also found it very close to the apex of the maxillary first molar.^8^ We used these data to design the external traction hooks in a way that the line of action of force passes above the apex of the maxillary first molar. 

## Based on your large experience with surgery, which are the advantages of distraction osteogenesis when compared with conventional orthognathic surgery to treat craniofacial deformities? (Eduardo Franzotti)

Distraction osteogenesis allows for large advancement and provides better stability when compared to conventional surgery.^9^^,^ 10 Because we do not need fixation plates to stabilize distraction, we have the possibility of modifying the osteotomy based on the deformity and on patient's anatomy. For instance, if the deformity is infraorbital, the surgeon can make a higher osteotomy and correct the infraorbital deficiency.

## In order to proceed with orthognathic surgery, orthodontists used to recommend patients to wait for the end of growth. In which situations growing patients could benefit from distraction? (Geórgia Lau)

Gradual distraction can be performed at any age, and growing patients with severe functional problems, such as breathing concerns, can benefit from it. Younger patients are not candidates for orthognathic surgery, as osteotomy and fixation screws can damage important structures, such as teeth. In addition, the effect on facial growth can be significant, since there is more scarring and fixation plates are used, which could also restrict growth. Conversely, during distraction, the healing of the bone is not dependent on fixation plates. 

## Severe mandibular deficiency present in Pierre Robin sequence usually features respiratory concerns, such as obstructive sleep apnea syndrome. At what time do you recommend distraction in those patients? (Lucio Maia)

Treatment of Pierre Robin patients is one of the greatest successes in the history of distraction osteogenesis. Patients can be operated as infants, and the procedure prevents tracheostomy in the event of respiratory obstruction. In the absence of functional problems, patients do not need early intervention. Whenever possible, it is good to wait until the end of facial growth. It should be recognized that even severe patients have some mandibular growth potential.

## If a recently trained orthodontist asks your advice on distraction osteogenesis or on how to be part of a craniofacial team, what would you tell him? (Eduardo Franzotti)

First, be the best orthodontist you can be and learn how to work as part of a team. Most of the care we provide is through a teamwork approach, even if we do not label it as such. We work on a daily basis with general dentists, pediatric dentists, periodontists, oral surgeons, etc. for the common good of our patients. Patients that require additional care, such as patients with cleft lip and palate and craniofacial anomalies, benefit from this teamwork approach. The difference is that we need to work not only with dental specialists, but also with members of other medical specialties, such as craniofacial surgeons, pediatricians, geneticists, speech pathologists, psychologists, etc. to provide the necessary multidisciplinary care needed by these complex patients. Thus, we need to get out of the "dental cocoon" and interact with other medical specialists. If you are part of the team, you will contribute with your expertise, and your expertise is as important as the one provided by any other member of the team. Be prepared to follow patients for a long time, remember you are the expert on facial growth and as such you will be consulted from infancy to adulthood. The relationships you develop with other team members, such as surgeons, will allow you to treat conditions you thought would be impossible to face. It will open your mind and will allow you to be a better orthodontist for your day-to-day patients. The long-term relationship that orthodontists develop with their patients makes them suitable for this challenge, and many times it is the orthodontist that patients seek for advice concerning treatment matters, even if the latter are not orthodontic in nature. Following patients in the long-term is one of the greatest joys you will receive as a result of your involvement with a craniofacial unit. Seeing a baby born with a difficult condition, helping him along the way and finally seeing him becoming a successful happy individual is one of the greatest rewards anyone can experience. So get involved!
